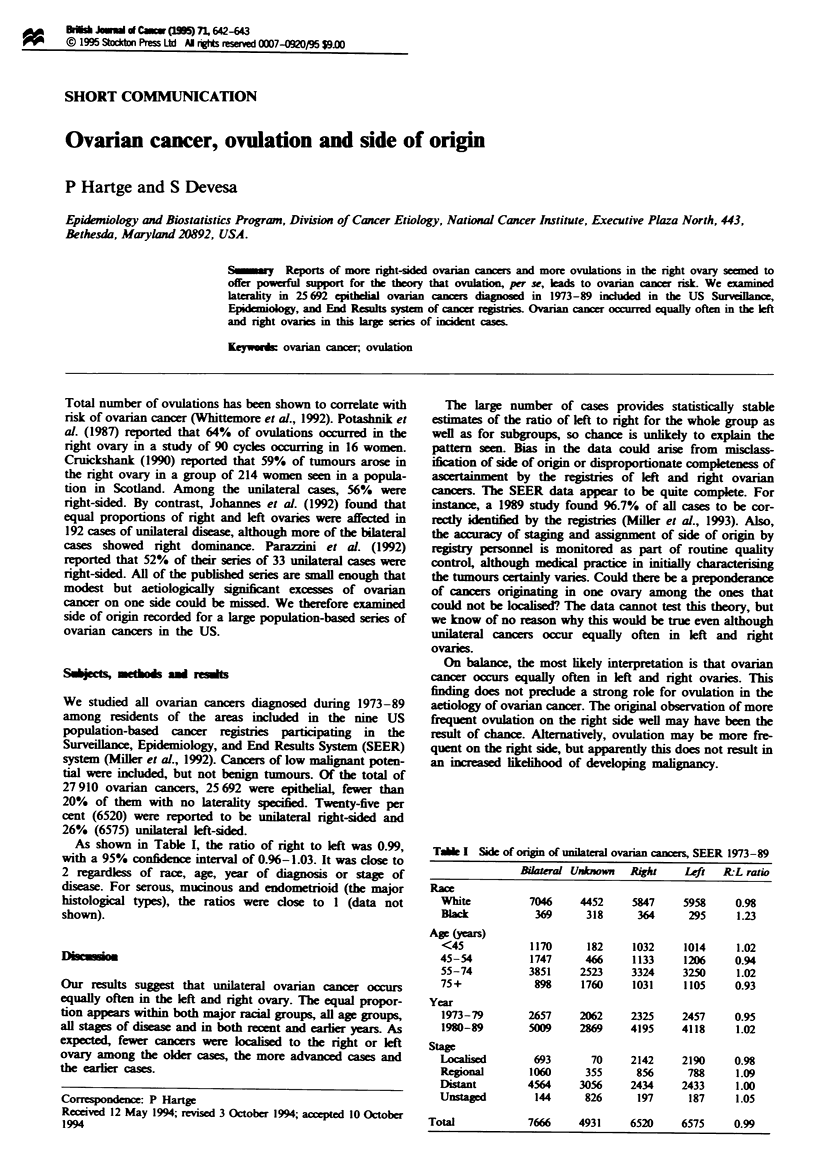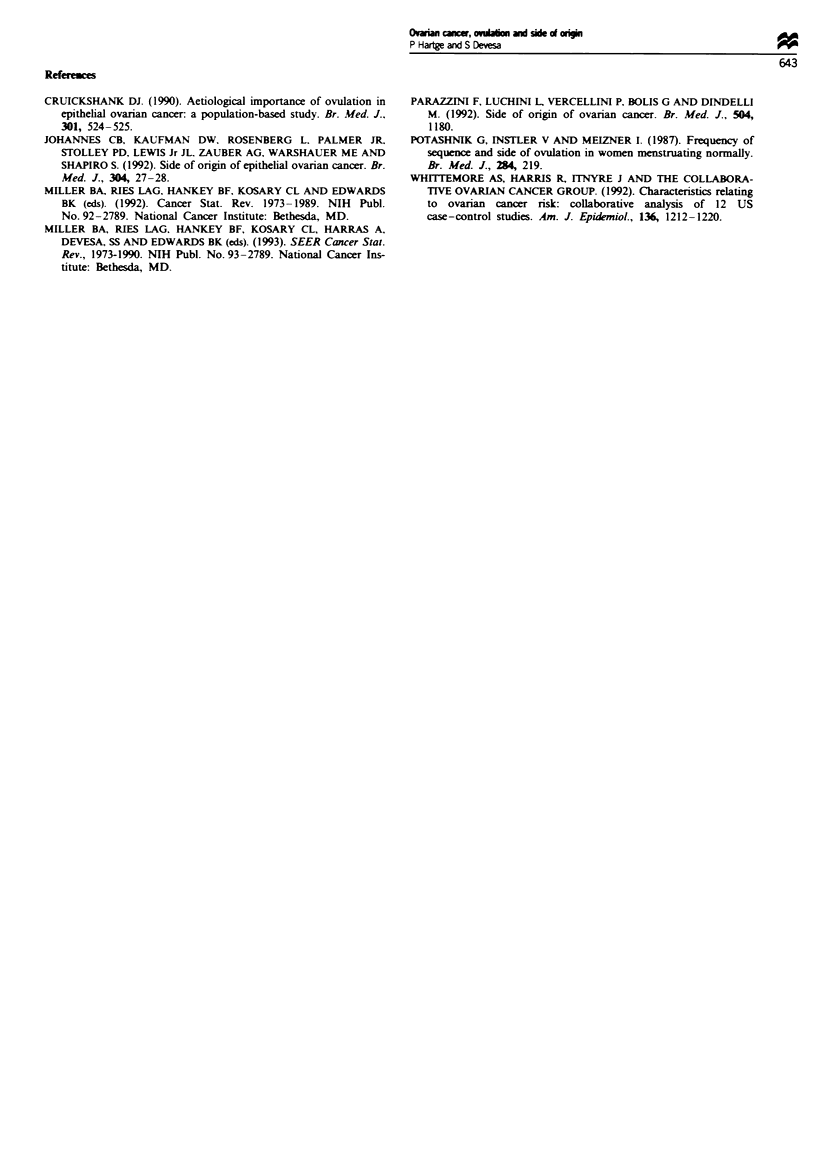# Ovarian cancer, ovulation and side of origin.

**DOI:** 10.1038/bjc.1995.126

**Published:** 1995-03

**Authors:** P. Hartge, S. Devesa

**Affiliations:** Epidemiology and Biostatistics Program, National Cancer Institute, Bethesda, Maryland 20892.

## Abstract

Reports of more right-sided ovarian cancers and more ovulations in the right ovary seemed to offer powerful support for the theory that ovulation, per se, leads to ovarian cancer risk. We examined laterality in 25,692 epithelial ovarian cancers diagnosed in 1973-89 included in the US Surveillance, Epidemiology, and End Results system of cancer registries. Ovarian cancer occurred equally often in the left and right ovaries in this large series of incident cases.


SHORT COMMUNICATION

### Ovarian cancer, ovulation and side of origin

P Hartge and S Devesa

*Epidemiology and Biostatistics Program, Division of Cancer Etiology, National Cancer Institute, Executive Plaza North, 443, Bethesda, Maryland 20892, USA.*

**Summary** Reports of more right-sided ovarian cancers and more ovulations in the right ovary seemed to offer powerful support for the theory that ovulation, *per se*, leads to ovarian cancer risk. We examined laterality in 25 692 epithelial ovarian cancers diagnosed in 1973-89 included in the US Surveillance, Epidemiology, and End Results system of cancer registries. Ovarian cancer occurred equally often in the left and right ovaries in this large series of incident cases.

**Keywords:** ovarian cancer; ovulation

Total number of ovulations has been shown to correlate with risk of ovarian cancer (Whittemore *et al.*, 1992). Potashnik *et al.* (1987) reported that 64% of ovulations occurred in the right ovary in a study of 90 cycles occurring in 16 women. Cruickshank (1990) reported that 59% of tumours arose in the right ovary in a group of 214 women seen in a population in Scotland. Among the unilateral cases, 56% were right-sided. By contrast, Johannes *et al.* (1992) found that equal proportions of right and left ovaries were affected in 192 cases of unilateral disease, although more of the bilateral cases showed right dominance. Parazzini *et al.* (1992) reported that 52% of their series of 33 unilateral cases were right-sided. All of the published series are small enough that modest but aetiologically significant excesses of ovarian cancer on one side could be missed. We therefore examined side of origin recorded for a large population-based series of ovarian cancers in the US.

### Subjects, methods and results

We studied all ovarian cancers diagnosed during 1973-89 among residents of the areas included in the nine US population-based cancer registries participating in the Surveillance, Epidemiology, and End Results System (SEER) system (Miller *et al.*, 1992). Cancers of low malignant potential were included, but not benign tumours. Of the total of 27 910 ovarian cancers, 25 692 were epithelial, fewer than 20% of them with no laterality specified. Twenty-five per cent (6520) were reported to be unilateral right-sided and 26% (6575) unilateral left-sided.

As shown in Table I, the ratio of right to left was 0.99, with a 95% confidence interval of 0.96-1.03. It was close to 2 regardless of race, age, year of diagnosis or stage of disease. For serous, mucinous and endometrioid (the major histological types), the ratios were close to 1 (data not shown).

### Discussion

Our results suggest that unilateral ovarian cancer occurs equally often in the left and right ovary. The equal proportion appears within both major racial groups, all age groups, all stages of disease and in both recent and earlier years. As expected, fewer cancers were localised to the right or left ovary among the older cases, the more advanced cases and the earlier cases.

The large number of cases provides statistically stable estimates of the ratio of left to right for the whole group as well as for subgroups, so chance is unlikely to explain the pattern seen. Bias in the data could arise from misclassification of side of origin or disproportionate completeness of ascertainment by the registries of left and right ovarian cancers. The SEER data appear to be quite complete. For instance, a 1989 study found 96.7% of all cases to be correctly identified by the registries (Miller *et al.*, 1993). Also, the accuracy of staging and assignment of side of origin by registry personnel is monitored as part of routine quality control, although medical practice in initially characterising the tumours certainly varies. Could there be a preponderance of cancers originating in one ovary among the ones that could not be localised? The data cannot test this theory, but we know of no reason why this would be true even although unilateral cancers occur equally often in left and right ovaries.

On balance, the most likely interpretation is that ovarian cancer occurs equally often in left and right ovaries. This finding does not preclude a strong role for ovulation in the aetiology of ovarian cancer. The original observation of more frequent ovulation on the right side well may have been the result of chance. Alternatively, ovulation may be more frequent on the right side, but apparently this does not result in an increased likelihood of developing malignancy.

**Table I** Side of origin of unilateral ovarian cancers, SEER 1973-89

| | *Bilateral* | *Unknown* | *Right* | *Left* | *R:L ratio* |
|---|---|---|---|---|---|
| Race | | | | | |
| White | 7046 | 4452 | 5847 | 5958 | 0.98 |
| Black | 369 | 318 | 364 | 295 | 1.23 |
| Age (years) | | | | | |
| <45 | 1170 | 182 | 1032 | 1014 | 1.02 |
| 45-54 | 1747 | 466 | 1133 | 1206 | 0.94 |
| 55-74 | 3851 | 2523 | 3324 | 3250 | 1.02 |
| 75+ | 898 | 1760 | 1031 | 1105 | 0.93 |
| Year | | | | | |
| 1973-79 | 2657 | 2062 | 2325 | 2457 | 0.95 |
| 1980-89 | 5009 | 2869 | 4195 | 4118 | 1.02 |
| Stage | | | | | |
| Localised | 693 | 70 | 2142 | 2190 | 0.98 |
| Regional | 1060 | 355 | 856 | 788 | 1.09 |
| Distant | 4564 | 3056 | 2434 | 2433 | 1.00 |
| Unstaged | 144 | 826 | 197 | 187 | 1.05 |
| Total | 7666 | 4931 | 6520 | 6575 | 0.99 |

Correspondence: P Hartge
Received 12 May 1994; revised 3 October 1994; accepted 10 October 1994

### References

CRUICKSHANK DJ. (1990). Aetiological importance of ovulation in epithelial ovarian cancer: a population-based study. *Br. Med. J.*, **301,** 524-525.

JOHANNES CB, KAUFMAN DW, ROSENBERG L, PALMER JR, STOLLEY PD, LEWIS Jr JL, ZAUBER AG, WARSHAUER ME AND SHAPIRO S. (1992). Side of origin of epithelial ovarian cancer. *Br. Med. J.*, **304,** 27-28.

MILLER BA, RIES LAG, HANKEY BF, KOSARY CL AND EDWARDS BK (eds). (1992). Cancer Stat. Rev. 1973-1989. NIH Publ. No. 92-2789. National Cancer Institute: Bethesda, MD.

MILLER BA, RIES LAG, HANKEY BF, KOSARY CL, HARRAS A, DEVESA, SS AND EDWARDS BK (eds). (1993). *SEER Cancer Stat. Rev.*, 1973-1990. NIH Publ. No. 93-2789. National Cancer Institute: Bethesda, MD.

PARAZZINI F, LUCHINI L, VERCELLINI P, BOLIS G AND DINDELLI M. (1992). Side of origin of ovarian cancer. *Br. Med. J.*, **504,** 1180.

POTASHNIK G, INSTLER V AND MEIZNER I. (1987). Frequency of sequence and side of ovulation in women menstruating normally. *Br. Med. J.*, **284,** 219.

WHITTEMORE AS, HARRIS R, ITNYRE J AND THE COLLABORATIVE OVARIAN CANCER GROUP. (1992). Characteristics relating to ovarian cancer risk: collaborative analysis of 12 US case-control studies. *Am. J. Epidemiol.*, **136,** 1212-1220.